# Implant Therapy in Patients With Neurodegenerative Diseases—A Scoping Review

**DOI:** 10.1111/clr.70080

**Published:** 2026-01-19

**Authors:** Lysandre David, Sabrina Maniewicz, Najla Chebib, Gabriel Gold, Murali Srinivasan, Frauke Müller

**Affiliations:** ^1^ Division of Gerodontology and Removable Prosthodontics, University Clinics of Dental Medicine University of Geneva Geneva Switzerland; ^2^ Department of Rehabilitation and Geriatrics University of Geneva Geneva Switzerland; ^3^ Clinic of General‐, Special Care‐ and Geriatric Dentistry, Center for Dental Medicine University of Zurich Zurich Switzerland; ^4^ Center of Excellence in Precision Medicine and Digital Health, Department of Physiology, Faculty of Dentistry Chulalongkorn University Bangkok Thailand

**Keywords:** Alzheimer's disease, dementia, dental implants, geriatric dentistry, Huntington's disease, Morbus Parkinson, neurodegenerative diseases, Parkinson's disease

## Abstract

**Objectives:**

The increasing longevity of populations has resulted in a growing number of older adults requiring prosthodontic care, including those with neurodegenerative diseases (NDs). Neurodegenerative diseases pose significant challenges to prosthodontic care, and it remains unclear whether implant therapy in this population achieves outcomes comparable to those observed in the general older population.

**Materials and Methods:**

A first systematic review was reframed to a scoping review using a PCC framework with Population (individuals with neurodegenerative diseases, who were partially or completely edentulous), Concept (implant therapy, planning, placement and maintenance, and any reported complications) and Context (dental and geriatric care settings).

**Results:**

The literature search identified 634 studies or case reports, of which none fulfilled the inclusion criteria. Seven papers (1 retrospective study, 2 prospective studies and 4 case reports) outside the inclusion criteria reported on patients with neurodegenerative diseases receiving implants, which suggest that dental implants seem to offer initial benefits in improving chewing efficiency, the quality of life and weight gain, especially in Parkinson's disease (PD) patients. However, their suitability for patients with advanced ND is uncertain.

**Conclusion:**

Although high‐level evidence on implant survival and success in patients with neurodegenerative diseases is lacking, the limited available evidence offers promising indications of reasonably successful implant treatments in early‐stage cases. However, continuous monitoring of disease progression, oral health and denture management is crucial to retrofit the restoration when necessary.

## 1 Introduction

People today are living longer and retain their natural dentition until later in life. Prosthodontic treatments are therefore ever more often provided for old and very old patients, whose orofacial system is characterized by signs of aging, along with an increasing prevalence of chronic diseases and frailty (MacEntee et al. 2025). The risk of being affected by a degenerative disease is exponential and directly correlated to age. These progressive diseases will, with time, have an increasing impact on patients and their oral health. To date, there is no cure, although recent drug developments are promising.

Neurodegenerative diseases such as Alzheimer's disease (AD), the leading cause of dementia, or Parkinson's disease (PD), a leading cause of movement disorders, affect more and more people worldwide (Zhang et al. 2021). Reports suggest that up to 46 million people were affected by Alzheimer's and related diseases worldwide in 2015, with up to 4.6 million new cases per year (Ferri et al. 2005; Alzheimer's Disease International 2015). PD was reported to affect 6.1 million patients worldwide in 2016 and its prevalence has increased by 21.7% between 1990 and 2016 (Marras et al. 2018). In contrast to AD, where women are more affected, PD affects men and women equally (Zirra et al. 2023). Huntington's disease (HD) is a neurodegenerative disease that affects both movement and cognition.

People with AD experience a decline in their ability to learn new information, perform daily tasks, use instruments like a toothbrush or a comb, and orientate themselves in time and space. As the disease progresses, impairments in executive function and worsening apraxia lead to increased dependence for daily care and hygiene. Marked progress in the dementia field over the past decades has led to more accurate diagnoses and earlier detection thanks to the wider use of cognitive screening tools such as the Mini‐Mental State Examination (MMSE) (Folstein et al. 1975), the Montreal Cognitive Assessment (MoCA) (Nasreddine et al. 2005), or the clock drawing test (Hazan et al. 2018; Shulman 2000), improvements in neuroimaging and the development of cerebrospinal fluid and plasma biomarkers.

An involuntary weight loss may accompany neurodegenerative decline, which, within a dental context, may result in an unusually rapid loss of retention of a complete upper prosthesis (Brubacher et al. 2004).

The exact cause of PD is unclear; however, both genetic and environmental factors are thought to play a role in the pathogenesis. Common clinical features are tremor, rigidity, and bradykinesia (Anglade et al. 1997; Hayes et al. 2019). As the head and neck muscles are concerned, patients present with involuntary mandibular and head movements which may render the dental treatment difficult. Incompetence of the lips and saliva pooling due to dysphagia induced by bradykinesia of the pharyngeal muscles may lead to drooling (22%), but 67% of patients rather present with xerostomia. Most patients present with loose dentures which may accelerate the symptoms (Anastassiadou et al. 2002). Routine oral hygiene measures become increasingly difficult as the disease progresses.

Oral health is impaired in patients with neurodegenerative diseases when compared to healthy people of a similar age. AD patients present with poor oral hygiene, fewer natural teeth, a high caries index, and periodontal disease (Aragón et al. 2018). Often, carious lesions remain untreated due to a lack of access to oral care or because of care resistance and decreased compliance (Ellefsen et al. 2009). The chewing efficiency and bite force are significantly reduced, and the use of removable dentures is low (Miura et al. 2003; Syrjala et al. 2012; Taji et al. 2005). Currently, there is limited to no information available regarding the prevalence of dental implants in these patient populations. Therefore, this review aimed to identify and map the existing evidence on the use of dental implants in patients with neurodegenerative diseases, describing reported indications, outcomes, and knowledge gaps.

## 2 Material and Methods

The protocol for this scoping review was registered at the International Prospective Register of Systematic Reviews (PROSPERO) under the number CRD420250653751. At first this article was planned as a systematic review, but due to the limited number of eligible studies and insufficient data for performing a meta‐analysis, the review was reframed for a scoping review. The framework was changed from a PICO format to a PCC framework (Population—Concept—Context) usually used for scoping reviews (according to PRISMA‐ScR guidelines). This article will still display both frameworks used for transparency of the methodological change.

The present scoping review concerns the use of implant treatment in patients with neurodegenerative diseases. The PICO format for the literature search is (P) Population with a minimum age 75, partially or completely edentulous, (I) having received dental implants and restored with fixed (IFDP) or removable dental prostheses (IOD). (C) Comparison was done with neurodegenerative diseases. (O) The outcomes assessed were survival, failures, and complications. The PICO focus question, eligibility criteria, and search strategy are shown on Table 1.

**TABLE 1:** PICO focus question, criteria for inclusion, sources of information, search terms, search strategy, search filters, and search dates.

Focus question	In edentulous geriatric patients (aged 75 years and over) treated with dental implants, what are the implant survival‐, biological complication, and technical complication‐, rates when comparing older adults with different neurocognitive disorders?	
Criteria	Inclusion criteria	Dental implants placed in the completely and partially edentulous human patients Implant supported fixed and removable prostheses Studies must specify the study design, number of patients, number of implants placed and failed, time of loading and number of dropouts Implant type: two‐piece, micro‐rough surface, solid screws Patients must have been clinically examined during recall
	Exclusion criteria	Age < 75 years Zygomatic, pterygoid implants Post–loading follow‐up < 12 months Implant diameter less than 3 mm Sample size of less than 10 cases
Information sources	Electronic databases	PubMed, Embase, the Cochrane Central Register of Controlled Trials (CENTRAL)
	Journals	All peer reviewed dental journals available online in databases: PubMed, Embase, CENTRAL
	Others	Popular online internet search engines (e.g., Google, Yahoo, etc.), Online internet research community websites (https://www.researchgate.net/), reference crosschecks, personal communications, hand‐searches, etc.
Search Terms (*PICO*)	Population	**#1**: **MeSH**—(Elderly Adults) OR (Partially Edentulous) OR (Fully Edentulous) OR (Completely Edentulous) OR (Partially Edentulous Maxilla) OR (Fully Edentulous Maxilla) OR (Completely Edentulous Maxilla) OR (Partially Edentulous Mandible) OR (Fully Edentulous Mandible) OR (Completely Edentulous Mandible) OR (80+ Aged) OR (75+ Aged) OR (75+ Aged) OR (Older Patient) OR (Aged Patients)
	Intervention or exposure	**#2**: **MeSH**—(dental implantation, endosseous) OR (dental implants) OR (dental prosthesis, implant supported) OR (Overdentures) OR (Removable dental prostheses) OR (fixed dental prostheses)
		**#3**: **All fields**—(dental implantation*) OR (dental implant) OR (implants) OR (implant supported fixed dental prostheses) OR (implant supported overdentures) OR (Removable dental prostheses*) OR (Overdentures) OR (Implant supported Overdentures) OR (Implant assisted Overdentures)
	Comparison	**#4**: **MeSH** –(neurocognitive disorder) OR (unipolar depression) OR (depressive disorder) OR (Alzheimer's disease) OR (dementia) OR (frontotemporal dementia) OR (mixed dementias) OR (dementia, vascular) OR (Creutzfeldt‐Jakob Syndrome) OR (Korsakoff syndrome) OR (hydrocephalus, normal pressure) OR (Parkinson disease) OR (Huntington disease)
	Outcome	**#5**: **MeSH**—(Survival) OR (survival rate) OR (survival analysis) OR (implant survival) OR (dental implant survival rate) OR (peri‐implantitis) OR (periimplant mucositis) OR (peri‐implant mucositis) OR (treatment failure) OR (prevalence) OR (mandibular implants failure rate) OR (maxillary implants failure rate) OR (success rate) OR (failure rate) OR (crestal bone loss) OR (periimplant bone loss) OR (bone loss) OR (periodontal conditions) OR (peri‐implant conditions) OR (implant success rates) OR (implant failure rates)
		**#6**: **All fields**—(dental implant success rate) OR (dental implant failure rates) OR (biological complications) OR (OHIP) OR (OHRQoL) OR (prosthetic technical complications) OR (prosthetic mechanical complications) OR (prosthetic maintenance)
Filters	Language	Not applied
	Species	Humans [MeSH]
	Ages	75+ Aged [MeSH]
	Journal categories	Dental journals
Search Builder	Search combination	#1 AND (#2 OR #3) AND #4 AND (#5 OR #6) AND Humans AND Aged
Search query as performed in the electronic databases	PubMed (Medline)	
	Embase	
	CENTRAL	
Search dates	January 1980–12.03.2025	Last confirmatory online final search was performed on 12.03.2025. No further online searches were performed after this date

According to the initial inclusion criteria, studies were included, when reporting on dental implants placed in completely and partially edentulous humans, restored with fixed (IFDP) or removal dental prostheses (IOD). Inclusion criteria also comprised the mention of a clinical examination, and the use of two‐piece solid screws with a micro‐rough surface. Exclusion criteria comprised patients < 75 years of age (as the specific category of “older adults” was evaluated with this study), zygomatic and pterygoid implants, a post loading follow‐up < 12 months, and an implant diameter of less than 3 mm and a sample size of less than 10 cases.

Once the methodology was changed and the article became a scoping review, a PCC was defined (according to the PRISMA‐ScR guidelines) as follows:

P—Population: Individuals with neurodegenerative diseases (e.g., Alzheimer's disease, Parkinson's disease) who are partially or completely edentulous and candidates for implant‐supported rehabilitation.

C—Concept: Implant therapy—planning, placement, and maintenance of dental implants; prosthetic rehabilitation (fixed or removable); and any reported indications, complications related to the management of neurodegenerative conditions.

C—Context: Dental and geriatric care settings, including hospitals, university clinics, dental clinics—where implant therapy is provided to older adults or patients with special care needs.

Where information was missing, the authors were contacted. Data charting was performed by two independent researchers (LD and FM), and mismatches were resolved by discussion. Author, year, and country; the study design; the investigated condition; the number of patients with implants and their mean age; the observation period; the number of implants placed and failed; time of failure; implant and attachment system; and calculated survival rates for implants and RPDs; number of patients and dropouts; number of implants placed/failed; and failure charted from the studies where available.

## 3 Results

**FIGURE 1:**
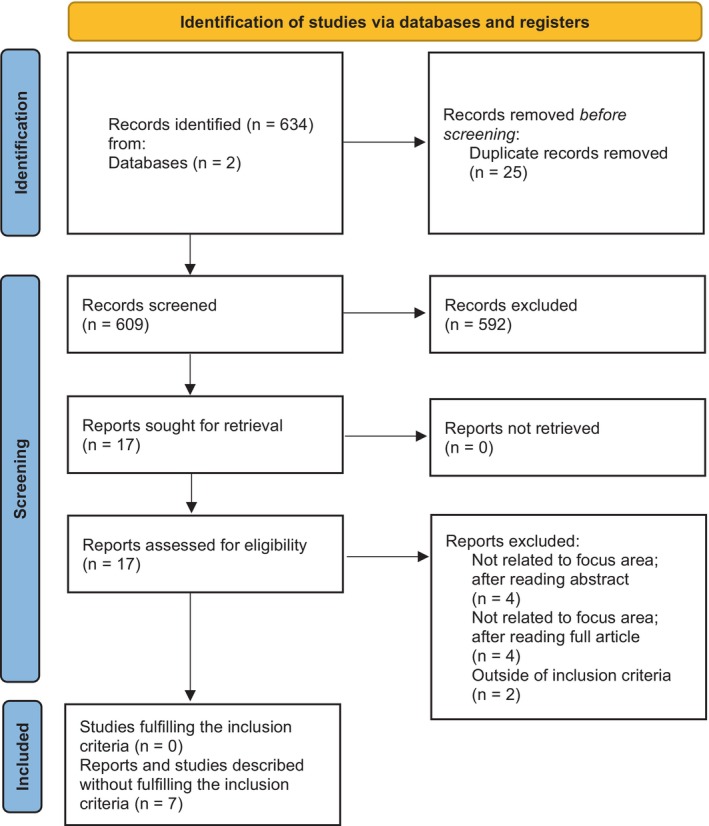
Article selection flowchart. Source: Page MJ, et al. BMJ 2021; 372: n71. https://doi.org/10.1136/bmj.n71.

The search revealed 634 articles (PubMed = 606 and Embase = 28). After removing the duplicates, 609 articles remained for the screening of the titles, of which 592 were excluded because they were not related to the focus area. Seventeen articles were downloaded for full‐text analysis. No study fulfilled the inclusion criteria (Figure 1). Finally, all reports mentioning clinically examined patients with neurodegenerative diseases and at least some patients who had implants placed within the context of a study or case report were described, even if they did not fulfill the initially defined inclusion criteria. In total, seven articles were included. This included 1 retrospective study, 2 prospective studies, and 4 case reports. Due to the heterogeneity of these reports, their primary findings were summarized descriptively for this review (Table 2).

**TABLE 2:** Studies and case reports including implant placement and prosthetic reconstruction in patients with neurodegenerative disease.

First author	Publication year	Country	Study type	Investigated condition	Number of patients with implants and neurodegenerative disease	Mean age in years	Observation period in months	Total number of implants placed or present	Implant system	Total number of implants failed in the study	Attachment system	Number of implants survived	Calculated implant survival rate	Time of failure	Survival RDP	Main outcomes of the study
Lopez Jimenez	2003	Spain	Retrospective	18 special care patients, including 3 cases of early‐stage dementia	3	34.7	66.5	67	N/A	4	All fixed reconstructions	63	94.4%	Before loading	100%	The survival rate in the 3 patients suffering dementia was 100% without a mention of the specification of the follow‐up time for these 3 patients. Age was not reported (Lopez‐Jimenez et al. 2003)
Tokumoto	2024	Japan	Case report	Parkinson's disease and dementia	1	81	240	7	Branemark	2 fractures, 1 submucosal sleeper	Locator	4	3/7 failed	7 years and 18 years	IOD no longer used after 5 years	Replacing a fixed implant prostheses by an implant supported overdenture on 5 implants with locator attachments (only 2 had Nylon inserts) to facilaitate denture management. Overdentures could no longer be worn after hospitalization and healing caps were installed, causing perpetual mucosal ulcers (Tokumoto et al. 2024)
Heckmann	2000	Germany	Prospective	Parkinson's disease	3	75.7	18 and 42	9	ITI Straumann solid screw	0	Non‐ridig telescopic copings	9	100%	N/A	100%	3 edentulous patients had received overdentures. Perceived chewing efficiency was improved in all 3 patients and the body weight increased on average 2.2 kg after denture delivery (Heckmann et al. 2000)
Chu	2004	Hong Kong	Case report	Parkinson's disease and mild stroke	1	83	12	4 (only 2 loaded)	Nobel Biocare TiUnite	0	2 magnets	4	Yes	N/A	Yes	Mandibular overdenture on x implants with magnetic attachments proved successful, 1 magnetic keeper became loose, the patient was satisfied and the family noticed improvements in the selection of foods (Chu et al. 2004)
Packer	2009	UK	Prospective	Parkinson's disease	9	63	3 and 12	28 loaded, plus 4 sleeers, and 6 not used	Astra Tech	5	Fixed and removable	23	82% (85% maxilla; 81% mandible)	Stage II surgery, thereafter no more failures	100%, 2 difficulties to remove denture, 1 fracture overdenture	Cohort of PD patients provided with either fixed or removable prostheses, significant improvements in eating and satisfaction with prosthesis. OHRQoL indicated gradual improvement of oral well being over the observation period. 1 patient died before the 12 months follow‐up (Packer et al. 2009)
Deniz	2011	Turkey	Case report	Huntington's disease and dementia	1	67	12	2	Straumann tissue level	0	Ball attachments	2	Yes	N/A	Yes	Surgery under sedation, because of involuntary movements. The overdenture was stable and the patient perceived a significant improvement in chewing (Deniz et al. 2011)
Jackowski	2001	Germany	Case report	Huntington's disease	1	56	12	2	Straumann TPS screw	0	2 implant bar	2	Yes	N/A	Yes, but bar unscrewed after 2 months	An overdenture retained by a 2‐implant bar improved significantly the chewing efficiency in this edentulous Huntington's disease case. Implant placement was performed under general anesthesia, The bar became unscrewed after 2 months, probly to continuous tongue movements, but could be reinserted unevenetfully (Jackowski et al. 2001)

Lopez‐Jimenez reported on a retrospective study on special care patients with fixed implant restorations, of whom 3 were diagnosed with early‐stage senile dementia (Lopez‐Jimenez et al. 2003). Age, implant type, type of restoration, and observation period were not specified; however, none of the three patients experienced implant failures.

Two prospective clinical studies including 3 and 9 patients with ND reported on dental survival rates, respectively. Heckmann et al. (2000) reported on three edentulous Parkinson's patients who received implant overdentures (IODs). The patients experienced improved chewing efficiency and gained an average of 2.2 kg after receiving their dentures. All implants survived the 3.5‐year observation period (Heckmann et al. 2000). A second prospective study from the United Kingdom included 9 Parkinson's patients and followed them for 1 year (Packer et al. 2009). Twenty‐eight implants were loaded with fixed or removable prostheses, 4 implants were not loaded, and 6 other implants were not used for the restorations. All implant failures were early, resulting in a survival rate of 82%. All participants reported being satisfied with the prostheses and experienced significant improvements in eating. The OHRQoL indicated a gradual improvement of the oral well‐being.

## 4 Discussion

No study fulfilled the strict inclusion criteria which are usually required for a systematic review on implant treatment. Only small prospective cohorts with PD were investigated, and no prospective study on dementia or HD patients was identified. As a lack of high‐level evidence was expected, all studies and case reports on patients with neurodegenerative diseases and dental implants were identified from the title screening process onwards. A possible reason for the lack of evidence might have been the too restrictive inclusion criteria. Another one is the cutoff age of exclusion of all studies including patients < 75 years of age. As mentioned previously, ND can affect patients from a younger age, but the evaluation of “older adults/geriatric patients” was what was looked at.

The lack of high‐level evidence like RCTs and the poor reporting in the few identified studies is certainly a weakness of this review. For instance, periodontal pocket depth around implants, marginal bone loss, plaque index, bleeding on probing, and implant mobility were all indexes that would be relevant, but none of the studies reported these outcome measures. The lack of high‐quality data seems at first a weakness of this review, but when reading the smaller studies and certainly the case reports, it becomes clear that it may indeed be a strength, in that the absence of evidence is a first pertinent information on the topic. Given the special characteristics of the dental care of patients with neurodegenerative diseases, it should not be surprising that dental implants are uncommon. In geriatric care, the classical success criteria for implants fall short (Buser et al. 1990). Denture management, the ability to perform effective oral hygiene measures, and the absence of injury from implant components may be equally pertinent for a successful implant restoration. The geriatric context has led to the introduction of the “back‐off” strategy, which recommends the simplification of an implant restoration along with the functional decline of an aging and frail individual (Müller and Schimmel 2016). In this concept, fixed restorations, which the patient no longer manages to adequately clean, are replaced by removable prostheses, and even in the latter, the retention mechanism may need to be simplified from a bar to a stud attachment to facilitate autonomous denture management. In the final stages of life, the attachment may even be unscrewed and replaced by a healing cap.

Although for different reasons, neurodegenerative diseases like dementia, PD, or HD all weaken motor coordination and muscle strength, with the peri‐oral and chewing muscles also being affected. Consequently, the chewing function is compromised (Miura et al. 2003) but can be improved by prosthodontic means (Campos et al. 2018). The disease‐related tremor in PD patients further complicates denture use and management. The retention of a conventional complete denture is achieved via occlusion, physical suction, and muscle control; hence it can become compromised when motor coordination weakens. Retaining a mandibular complete denture with inter‐foraminal implants replaces the above‐mentioned retention mechanisms. A well‐retained and functional prosthesis may contribute to the patient's well‐being and nutritional state. Heckmann and colleagues recorded an average weight gain of 2.2 kg in all 3 Parkinson's patients after an average of 35 weeks with the novel IOD (Heckmann et al. 2000). This protective weight gain is welcome in any geriatric patient (Weiss et al. 2008), but especially in patients with neurodegenerative diseases, as weight gain is associated with lower morbidity and mortality (Brubacher et al. 2004). In edentulous patients, the replacement of a conventional complete denture by a 2‐IOD leads to a significant increase in masticatory efficiency (van Kampen et al. 2004). Even if this measure alone does not lead to a healthier diet and thus to a better nutritional state in people living at home, it does allow a broader selection of foods (Chu et al. 2004; Hamdan et al. 2013). It may further avoid mixing meals for chewability, thereby rendering the dishes more appetizing. Chewing activity has further advantages, as it slows the chewing muscle atrophy and seems to have a positive effect on cognition and the conversion of MCI to AD (Kim et al. 2023; Müller et al. 2012; Takeuchi et al. 2015). In animal experiments, feeding a soft diet decreased dopamine release in the hippocampus and impaired learning ability and memory in AD model rats in comparison with rats fed a hard diet (Kushida et al. 2008). Packer et al. reported on a significant improvement in eating and satisfaction with the prostheses in their PD patients (Packer et al. 2009). In Parkinson's patients, the stabilization of a prosthesis may have another positive effect: the involuntary mandibular and peri‐oral muscle activity may be calmed, as an unstable denture serves as a trigger for mandibular dyskinesia (Wächter et al. 1996). Given the above‐mentioned functional improvements and the positive effect on well‐being and Oral Health‐Related Quality of Life, the use of implants in the restorative treatment of patients with neurodegenerative diseases seems indeed tempting.

However, despite all these advantages, implant therapy in patients with neurodegenerative diseases also has a downside, which refers to the progressive nature of the disease and the current lack of a cure. In the advanced stage of the disease, a decrease in independence regarding denture handling and oral hygiene is to be expected. Care‐resistant behavior is frequently reported in advanced‐stage AD patients. Another problem is that caretakers are insufficiently trained with regard to oral health in general, and dental implants in particular. In a survey in Japanese retirement homes, half of the surveyed care facilities stated that they were unable to recognize implants and did not know how to care for them (Kimura et al. 2015). However, in a recent French survey on the oral health of persons aged 90 years or over, only 2 out of 90 participants had implants (Rosa et al. 2020). One in 5 long‐term care facilities in Japan reports having residents with implants (Kimura et al. 2015). Although the implant prevalence is low, it will increase when the baby boomer generation becomes older and dependent on care.

With the progression of the neurodegenerative disease, denture use may become problematic. A prospective study from Japan related the frequency of denture use to the patient's cognitive state and found that only one in three severely demented patients is using his/her denture (Taji et al. 2005). A simple test may serve to evaluate the patient's capacity for denture management, the prosthesis presentation test. Here, the denture is presented reversed, and it is observed whether the patient can invert and insert it correctly. In a clinical study, 21 of 86 hospitalized geriatric patients were unable to turn the prosthesis correctly, which was significantly correlated with their functional independence measure (Srinivasan et al. 2024). Other reasons for not wearing the prostheses include radiation or chemotherapy‐induced mucositis, or simply a lack of motivation. Both implants and attachments can pose a risk of injury, especially in dentitions where occlusal support is no longer guaranteed by pairs of occluding teeth. Furthermore, like any hard object in the mouth, implants become colonized with biofilm, which, if oral hygiene is inadequate, may cause aspiration pneumonia (Sjögren et al. 2016).

While implants can be a blessing in the early stages of neurodegenerative diseases by improving denture stability and chewing function, they can become problematic in their advanced stages. The expected benefits from the treatment must outweigh the invasiveness and potential risks at a later stage. Close monitoring of implant patients is necessary to timely “retrofit” the prosthesis. Where possible, alternative treatment strategies may comprise the medium‐term preservation of compromised abutment teeth, which provide temporary mechanical retention and therefore facilitate the adaptation to the dentures. Another alternative to increase denture retention is the prescription of denture adhesives when conventional denture manufacturing protocols are no longer sufficient. The advantage of the latter strategy is that it is non‐invasive, not dependent on the general health status of the patient, and entirely reversible.

Future research should prioritize flexible and reversible treatment concepts that can be adjusted as patients' functional abilities decline.

## 5 Conclusion

This scoping review leaves a lot of questions unanswered. While high‐level evidence on implant survival and success in patients with neurodegenerative diseases is lacking, the few existing reports provide encouraging support for reasonably successful implant treatments, particularly in early‐stage cases. The functional and nutritional benefits may outweigh the potential risks, particularly for edentulous patients with unstable prostheses who have been diagnosed with PD. However, continuous monitoring of disease progression, oral health, and denture management is crucial.

## Author Contributions

Lysandre David: design of the study, literature search, data charting and interpretation, manuscript preparation, figures and tables, critical review of the final manuscript. Murali Srinivasan: search strategy, statistical summarization, interpretation of results, and critical review of the final manuscript. Najla Chebib, Sabrina Maniewicz, Gabriel Gold: interpretation of the results and critical review of the final manuscript. Frauke Müller: design of the study, data charting and interpretation, final review of the manuscript. All authors are accountable for all aspects of the work.

## Conflicts of Interest

The authors declare no conflicts of interest.

## Supporting information


**Data S1:** clr70080‐sup‐0001‐supinfo.pdf.

## Data Availability

Data sharing not applicable to this article as no datasets were generated or analyzed during the current study.
